# Physical and Mechanical Performance of Mortar with Rice Husk Ash and Sugarcane Bagasse Ash as Partial Cement Replacement

**DOI:** 10.3390/ma18204758

**Published:** 2025-10-17

**Authors:** Jyoti Rashmi Nayak, Małgorzata Gołaszewska, Jerzy Bochen

**Affiliations:** 1 Department of Civil Engineering, GITAM (Deemed to be University), Hyderabad 502329, India; jnayak@gitam.edu; 2 Department of Building Processes and Building Physics, Faculty of Civil Engineering, Silesian University of Technology, 44-100 Gliwice, Poland

**Keywords:** rice husk ash, bagasse ash, Portland cement, mortar, partial replacement, physical and mechanical properties

## Abstract

Natural supplemental cementitious materials (SCMs) with pozzolanic qualities, such as rice husk ash (RHA) and sugarcane bagasse ash (SCBA), are a promising alternative to the currently used SCMs that are becoming increasingly unavailable. This work presents a comprehensive comparative examination of their impact on mortar properties when OPC was partially replaced by RHA and SCBA. The percentage substitution of OPC with ashes was 0, 5, 10, and 15%. The air content, consistency, compressive strength, flexural strength, and shrinkage of the mortar were investigated primarily. Microstructural characteristics were analysed using porosimetry, MIP, and SEM photography. According to the study, up to 10% replacement of OPC with RHA or 15% with SCBA has the potential to be used as a partial cement substitute while maintaining good mechanical qualities. Mortars with up to 15% SCBA exhibited no significant change in compressive strength after 28 days or a decrease with <11%, while for 10% RHA, there was no difference in compressive strength or increase. Use of 5% RHA decreased shrinkage by 35%, while addition of 5% SCBA by 30%. Obtained results demonstrated the usefulness of SCMs in masonry mortars.

## 1. Introduction

Currently, great emphasis is put on environmental protection and sustainability in material design and research, and civil engineering is no different. One of the main issues when it comes to building materials is cement production, as it is currently a significant part of anthropogenic CO_2_ emissions due to the scale of the demand. One way of decreasing the carbon footprint of cement is to add supplementary cementitious materials, such as combustion waste (ashes), which not only decrease the amount of cement used but also provide a way of utilising waste that would otherwise need to be landfilled. The advantages of using such pozzolans, such as fly ash, rice husk ash, wood ash, and bagasse ash, as a partial replacement for Portland cement in the production of mortar and concrete are currently the subject of numerous studies in terms of the influence of factors, such as the duration of combustion, the temperature, the duration of cooling, and the grinding conditions on the characteristics of biomass ash, but also their effect on the properties of mortars that could lead to their more widespread use [1,2,3,4]. This article provides research on the use of two types of plant-based waste ashes: rice husk ash and sugarcane bagasse ash, as a replacement for cement, comparing their effects on different types of masonry mortars.

Rice husk ash has been used as a supplementary cementitious material (SCM) in recent decades because of its properties and it being one of the most widespread cultivated plants in the world. The most common application of rice husk itself is to use it as fuel, and after incineration, about 20% of its weight becomes rice husk ash (RHA), which can later be used in concrete as a supplementary cementitious material [5]. Rice husk ash (RHA) has a pozzolanic nature due to its high silica content, silica crystallisation phase, particle size, and surface area. It can be used successfully in concrete and mortar as a replacement for cement and silica fume while maintaining durability and strength characteristics [6,7]. At the application level, Coutinho [8] conducted a study of the effectiveness of RHA in replacing cement in hydraulic binders and mortars with good effects. Feng et al. [9] discovered that substitution of cement with RHA can improve the pore structure, resulting in smaller pores and, ultimately, its addition can result in increased compressive strength. The addition of the right proportion also plays an important role in strength. Ganesan et al. [10] concluded that 15% RHA as a cement replacement was the optimal replacement rate, taking into account compressive strength; however, a 10% RHA replacement also achieved a high compressive strength. Similarly, using RHA as a replacement for cement improved both durability and compressive strength according to research by Bui et al. [3], Zhang et al. [6], and Bezerra et al. [11], where the addition of up to 20% showed better mechanical properties in both mortar and concrete than that of the reference cement. Mahmud et al. [12] discovered that RHA used in concrete had higher strength and lower shrinkage than OPC concrete. Most of the studies focused mainly on the use of RHA as a replacement for cement in concrete, and these studies will help contribute more to different types of mortar with RHA. Furthermore, it was found that the maximum 15–20% RHA replacement rate provided the best results in terms of mechanical properties. Outside of mechanical properties, research on RHA properties does not provide a clear outline of the effect of RHA on properties of mortar [13,14]. While some research indicates an increased hydration rate and acceleration of hydration [15,16], a prolonged setting time and delayed hydration were also observed [17,18]. In case of workability, the effect was not unequivocal, as RHA can potentially decrease workability [16] or improve flowability [19], indicating that possibly a loss of ignition or fineness may be a deciding factor in terms of its effect on consistency. A very limited amount of information is available on the effect of RHA on shrinkage of cement mortars. Habeeb et al. [20] obtained results indicating that with the increase in fineness of RHA, the shrinkage of cement mortar increased. Huang and Ye [21] indicated that RHA could decrease autogenous shrinkage.

Sugarcane bagasse ash (SCBA) is a by-product of the combustion residue from extraction of sugar from the sugarcanes. It presents an attractive option for cement replacement in concrete applications due to its properties [22,23,24], as it contains significant amounts of amorphous silica (SiO_2_), alumina (Al_2_O_3_), and ferric oxide (Fe_2_O_3_) [25,26,27,28], which represent more than 70% of the constituents of SCBA, indicating that it can be used as a pozzolanic material. Furthermore, Moraes et al. [29] demonstrated that SCBA improves the microstructural and mechanical properties of mortars at addition with levels of 25% and 30% of plain cement. Similarly, in terms of durability, studies have shown that including SCBA improves resistance to sulphate attack and fire [30]. Modani [31] discovered that at 10% fine aggregate replacement with SCBA, the compressive strength was comparable to that of the control sample, but any further increase in the SCBA content resulted in a decrease in strength. Sajjad Ali Mangi [32] investigated the effect of replacing cement with SCBA at 0, 5, and 10% of concrete strength at 7, 14, and 28 days and concluded that replacement with 5% SCBA exhibited the better strength gain in comparison to reference samples due to the pozzolanic properties of bagasse ash. Batool et al. [33] conducted experiments utilising raw sugarcane bagasse ash in concrete, substituting it in proportions ranging from 5% to 30%. The scientists determined that the incorporation of SCBA improved workability, with a 10% substitution was deemed optimal for attaining elevated compressive, flexural, and tensile strengths. The optimal proportion for substituting cement by several studies determined that substituting SCBA with a cement content of 10–20% improved the mechanical characteristics of concrete [34,35]. The effect of SCBA on the consistency of cement mortars and concretes is generally stable, with the addition of SCBA decreasing workability [36,37,38]. The effect on setting and hydration is more varied, with some research indicating a delay of initial setting and prolonged hydration [39], while others indicate no change, or even acceleration of hydration [40]. As in the case of RHA, the effect of SCBA on shrinkage is not as widely researched; however, available research indicates the possibility of SCBA decreasing shrinkage [41,42].

Much research has been done on the uses of these materials in concrete work. However, although numerous research has been done on the topic of cement mortars, the amount of research into cement–lime mortars and mortars with plasticising, air-entraining admixtures, which are oftentimes used in practice, is limited, especially when it comes to comparisons between them. Cordeiro et al. [43] confirmed the pozzolanic nature of SCBA in lime mortars, with the fineness of the ash having a significant effect on the compressive strength. Similarly, the research by Malathy et al. [44] indicated the possibility of using RHA in lime mortars due to pozzolanic reaction. In terms of using RHA and SCBA in cement–lime mortars, the research by Cook et al. [45] showed the possibility of using RHA up to a 60% substitution rate of cementitious material in masonry units; however, its use in masonry mortars was not explored to a satisfactory degree.

Additionally, it should be noted that the number of studies on the shrinkage of mortars with SCBA and RHA is limited and often lacking in the systematic context of other tests, such as MIP. The novelty of this paper lies, therefore, in the comprehensive look at the effects of SCBA and RHA addition to the different types of renders and mortars (cement, lime–cement, and cement with air-entraining admixtures), allowing for comparisons between different mortars, on both often tested properties, such as strength or consistency, but also rarely tested ones, such as shrinkage and mercury intrusion porosimetry. This paper aims to increase the present knowledge on the topic by providing an extensive look at the properties of different mortars with SCBA and RHA, as well as to provide comprehensive comparative data for future work.

The main aim of this paper is to provide insight into the effect of RHA and SCBA used as a replacement for cement in different types of mortars based on masonry mortars, such as cement mortars, cement–lime mortar, and mortar with air-entraining plasticizer admixtures (APAs). This is an extended part of the research on the use of natural waste as building materials [46]. The study’s ongoing goal is to present the results of the physical, mechanical, and microstructural characterisation of mortars with partial replacement of cement by SCBA and RHA additives. We discuss the effects of adding 5%, 10%, and 15% RHA and SCBA (relative to cement weight) to cement compared to a control sample. In terms of fresh mortar properties, air content and consistency are studied, and for hardened mortar dry shrinkage, compressive and flexural strength at 7, 28, 56, and 90 days of age are evaluated in the study. Most of these properties are required by the BS EN 998-1 [47] for masonry mortars. Moreover, to observe the effect of additives on the microstructure of the cement matrix, scanning electron microscopic (SEM) and mercury intrusion porosimetry (MIP) are also analysed.

## 2. Materials and Methods

### 2.1. Materials

In the experimental program, supplementary cementitious ingredients were commercially available waste materials. The RHA used in this study was collected from a local landfill in Gujarat, India, and the SCBA from Chhattisgarh, India. CEM I 42.5 cement and hydrated lime were obtained from Lhoist Company in Poland. Dried natural river sand fractions of up to 2.0 mm were used as aggregate. The properties of these materials are presented in Table 1. On the basis of Novikov’s cone test, the water content in every mortar was chosen to provide a predefined consistency similar to that of a reference mortar. Air-entraining admixtures (APAs) are often used in everyday practice as a plasticising admixture and, therefore, have been included in the testing to better compare possible uses of RHA and SCBA for practical settings. Additionally, APAs are often promoted as a lime replacement for masonry mortars and plasters and, therefore, comparison between cement mortars with APA and cement–lime mortars is also important for possible practical applications.

**Figure 1:**
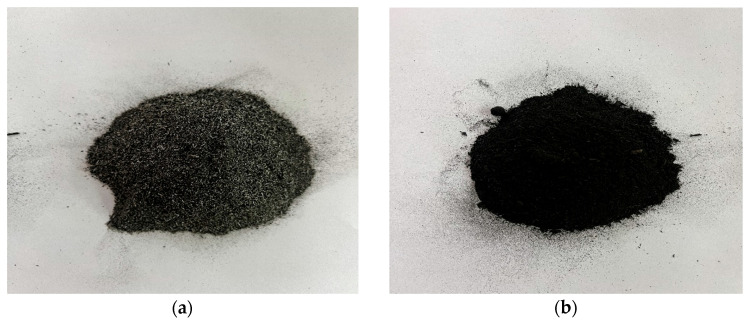
Photos of waste materials: (**a**) rice husk ash and (**b**) sugarcane bagasse ash.

**Figure 2:**
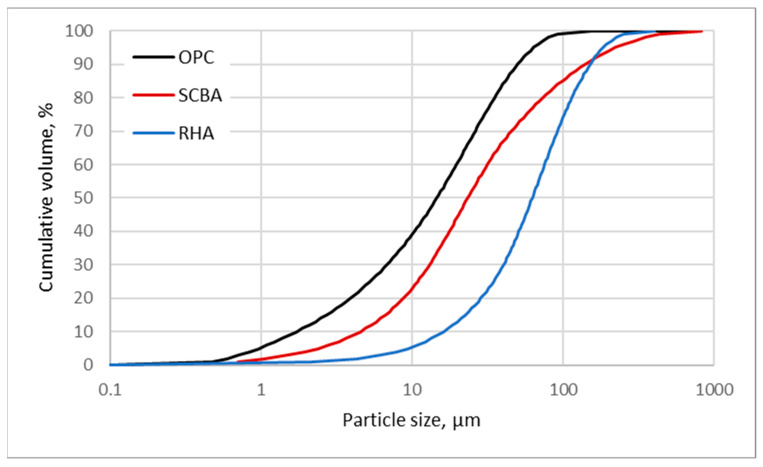
The particle size of cement and ashes used in the testing.

The natural biomasses used in this study are rice husk ash (RHA) and sugarcane bagasse ash (SCBA), as shown in Figure 1, with all the chemical and physical properties mentioned in Table 1 and Table 2, respectively. The particle size distribution, presented in Figure 2, was determined using a Bettersizer 2600 (3P Instruments GmbH & Co. KG, Odelzhausen, Germany) laser diffraction particle size analyser after drying the samples to dry mass. Materials were obtained from local cogeneration landfills. Due to the high carbon content, both of them were black (see Figure 1). The ashes used in the research were preprocessed by their producer. To obtain the ashes, the biological waste material was burned at 600–800 °C for one hour before cooling and being ground to small sized particles. Thermal treatment is commonly used for RHA and SCBA. RHA and SCBA are produced when rice husks and dry sugarcane residue are burnt at temperatures ranging from 500 to 800 degrees Celsius [48]. Most of the ashes consist of silicon dioxide (SiO_2_), and the presence of amorphous silica is considered sufficient. This is because it facilitates the chemical reactions that lead to the formation of hydrated calcium silicates, which play a crucial role in improving the mechanical strength of pastes, mortars, and concretes over a period of time [1,28].

RHA and SCBA are used as an excellent supplementary cementing material, and their fineness (Table 2 and Figure 2) can be improved by grinding. Ball or hammer mills are typically used to grind the product to a fine powder. The pozzolanicity of biomass ash is influenced by several aspects, such as composition, burning temperature, particle size, and chemical composition. It is crucial to take these factors into account [1,28,48,49,50]. These are collected after the calcination process and subjected to the dry grinding process.

In order to make mortar, ordinary Portland cement was partially replaced with 5%, 10%, and 15% RHA and SCBA. These proportions were chosen with practical application in mind—replacement of up to 15% of cement with pozzolanic material falls into the same category as cements CEM II/A. Although bioashes are not one of the recognised categories for main constituent of cement, the EN 197-1 [51] is being constantly updated to include more types of cements.

The cement–sand mixture was made in a 1:6 ratio, and the cement, hydrated lime, and sand were made in a 1:1:6 ratio. The amount of water was chosen based on the proper consistency for masonry mortars, namely, a 7–8 cm penetration depth, as measured by Novikov’s cone. Due to expected high water demand for ashes, as well as a high ratio of sand to cement, the w/b ratio was significantly higher than in the case of mortars used for testing of cement properties (usually in the range of 0.4–0.5 for cement mortars), or in concrete mixes (usually in the range of 0.3–0.5). Three reference samples with symbols C (cement mortar), CL (mortar with lime–cement), and CA (cement mortar with APA), and others with natural SCM, namely, SCBA(S) and RHA(R), were prepared. The air-entraining plasticiser admixture (APA) was taken with an amount of 0.5% to the volume of cement considered for all the APA samples. This amount was chosen experimentally, to achieve comparable consistency measured with the Novikov’s cone (described in detail in Section 2.2.1), and to fit in the middle range of its use suggested by the manufacturer. The mortar was mixed according to the mixing procedure mentioned in the EN 197-1 [51]. All mixtures, with and without ashes, were tested for air content and consistency as fresh properties, and shrinkage, flexural strength, and compressive strength as hardened mortar properties. Prismatic samples of size 40 × 40 × 160 mm^3^ were removed from the mould after 24 h and subsequently immersed in water for 2, 7, 28, 56, and 90 days prior to testing. The lime–cement sample underwent a curing process with water for a duration of 7 days, followed by curing in a humidity chamber maintained at a temperature of 20 ± 2 °C and a relative humidity of 60%. The proportions of the mortar mixture are given in Table 3. The samples were removed from the moulds after 24 h and then immersed in water until the specific ages required for each test were reached. If not indicated differently, the end result was calculated as a mean value of 3 samples.

### 2.2. Test Methods

#### 2.2.1. Properties of Fresh Mortar

For the fresh mortar properties, air content and consistency (by 2 methods) were tested.

The number of air voids in mortar is an important factor in reducing damage caused by freezing and thawing cycles, and the easiest way to provide information on the air void content is to measure air content in a fresh mortar by using the pressure method. This property is required for masonry mortar by the standard BS EN 998-1 [47]. Air content was measured using a standard porosimeter and the pressure method specified in PN-EN 413-2:2016-11 [52]. The apparatus comprises a 1 litre container for the mortar. The mortar is put in two layers, which are compressed with a tamper rod.

**Figure 3:**
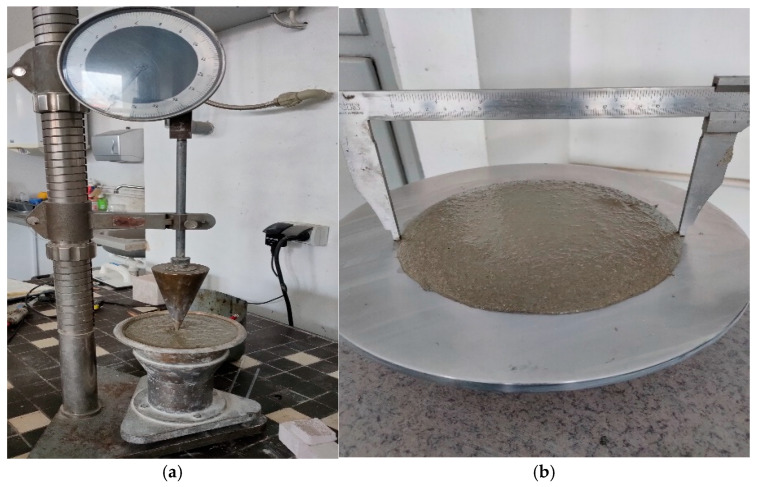
Consistency tests: (**a**) Novikov cone and (**b**) flow table.

Consistency is also required for masonry mortar according to the standard EN 998-1 [47]. Consistency was evaluated using two methods: the Novikov’s cone, primarily utilised in practical circumstances on building sites, and flow table analysis, which was established in a laboratory testing. Both approaches provided an average of three measurements. The experiment was carried out according to the guidelines of PN-85/B-04500 [53] using a Novikov’s cone apparatus (Figure 3a). The experiment involved the deposition of three levels of mortar mix into a conical container and its subsequent consolidation with the use of a tamper rod. The top layer of mortar was then smoothed, and a cone-shaped plunger with fixed weight was dropped from a predetermined height. The depth of penetration of the cone into the mortar was measured using a vernier scale. The second method was a flow table according to the standard EN 1015-3:2000 [54]. The Vernier scale was used to measure the change in the diameter of the paste in two directions to determine the flow diameter, as presented in Figure 3b.

#### 2.2.2. Properties of Hardened Mortar

For hardened mortar, flexural and compressive strength were tested, as well as drying shrinkage.

The flexural and compressive strengths of the mortars were evaluated according to the standard EN 1015-11:2001 [55]. Compressive strength is a recommended test for masonry mortars by the standard BS EN 998-1 [47]. After 24 h, cubic samples measuring 40 × 40 × 40 mm^3^ for compression and prismatic samples of 40 × 40 × 160 mm^3^ for bending were removed from the mould and subjected to a curing process in water for 2, 7, 28, 56, and 90 days. Both CL samples, with and without additives, were subjected to a 7-day water curing process. Subsequently, they were taken to a humidity chamber that was kept at a temperature of 20 ± 2 °C and a relative humidity of 50–60%. A controlled environment was required due to the addition of lime, which requires a dry atmosphere. The test result for both procedures yielded an average value based on three measurements in case of flexural strength and six samples in case of compressive strength. The results were analysed using the ANOVA statistical method, using Statistica software (version 13.3) from StatSoft (Krakow, Poland). ANOVA is the method of assessing the validity of observed differences in the results by means of analysis of variance. The significance level for the testing was set at *p*-value = 0.05, which is a generally accepted level of statistical significance.

Dry shrinkage was measured using the traditional method, in a Graff–Kaufmann device according to the EN 12617-4:2004 [56], which involves testing sample sizes of 40 × 40 × 160 mm^3^ at appropriate intervals of 3, 7, 21, and 28 days. The samples were kept with the apparatus in a climatic chamber at a temperature of 20 ± 2 °C and at 50–60% relative humidity. The 28-day mark was chosen, as in the cement mortars the majority of the shrinkage occurs in this timeframe [57]. Three samples were measured, and a mean value was calculated to obtain a result.

#### 2.2.3. Microstructural Parameters

The MIP and SEM examinations conducted at 28 days allowed for the determination and examination of the surface characteristics, form, and dimensions of the components. One sample was chosen for SEM testing and one for MIP testing. A 20 mm sample, without any visible fractures or irregularities, was chosen from samples with SCBA and RHA additions. Micrographs were obtained from TESCAN (Brno, Czech Republic), the Mira3 Oxford Instruments model microscope (Abingdon, UK). The tests were carried out on fracture surfaces that had been sprayed with a carbon conductive layer. The MIP measurements were made to determine the surface area of the pores, the dominant diameter of the pores, the tortuosity of the pores, open porosity, the apparent density, the true density, and the pore distribution. The Autopore IV 9500 instrument (Micromeritics Instrument Corporation, Norcross, GA, USA) was used to measure the amount of mercury that entered the pores in the size range of 6–4500 nm. The parameters assessed were calculated using the following constants: the contact angle between the sample and mercury was measured at −140 degrees, the surface tension of the mercury was determined to be 0.485 Nm^−1^, and the density of mercury was found to be 13.54 g/cm^3^.

## 3. Results and Discussion

### 3.1. Air Content Analysis

**Figure 4:**
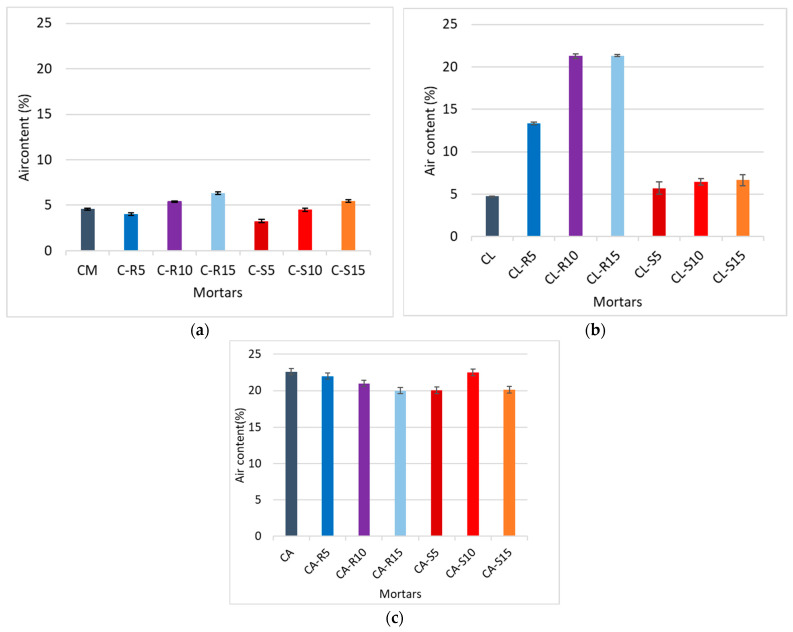
Air content value of (**a**) mortar with Portland cement, (**b**) mortar with Portland cement and lime, and (**c**) mortar with Portland cement and APA. Vertical bears indicate standard deviation.

Figure 4 illustrates the results of the air content tests. The study of air content is conducted due to the recommendations for masonry mortars and due to the fact that air-entraining admixtures were used in the mortar, with the aim of maintaining consistency. The air amount in lime–cement and cement mortars is noticeably reduced compared to APA mortars, as the air content for mortars with APA is between 20% and 25%, while the air content of cement mortars is 3–7%. This outcome was expected as a result of the effects of the air-entraining admixture.

In the case of cement mortar, with the addition of rice husk ash (RHA), the amount or air increases. A similar relationship can be observed in the case of cement–lime mortar; however, the effect is significantly more pronounced, with an increase of almost 20 percentage points in relation to the reference sample. Furthermore, the fineness of RHA is lower compared to that of SCBA, which can lead to a greater presence of air voids.

Interestingly, incorporation of SCBA into cement does not exhibit any notable alteration in air content in the case of both cement mortar and lime mortar. A similar trend is observed in a study by Awad et al. [4] investigating the impact of introducing 25% sugarcane bagasse ash into the mortar mix. In cement–lime mortar, air content values show a consistent increase at all levels of cement replacement with biomass ashes (SCBA and RHA) compared to the control sample. The increase in air content may be due to the irregular shape of the SCM’s grains [58]. The behaviour of biomass ashes in cement–lime samples appears to diverge from that of cement samples. The rationale for the increase in air content lies in the alteration of the water-to-cementitious material ratio, which, in turn, affects the need for water to facilitate the lubrication of cementitious particles. This change in water demand contributes to the observed increase in air content [59].

In APA cement mortar, the air content value is five times higher, as seen in Figure 4c, even with a lower water–binder ratio. The elevated air content observed in the presence of the APA can be attributed to its inherent air-entraining characteristics. These properties are activated by the foaming action of the admixture when it comes into contact with water. This phenomenon, in turn, induces greater porosity in the resulting hardened mortars. Consequently, this increased porosity contributes to reduced strength and adhesion, as evidenced by the outcomes presented in the results, which is consistent with other research in the area [60]. Substituting cement with biomass ash, such as RHA and SCBA, in the APA sample leads to a two percentage points reduction in air content. This can be attributed to the filler effect of SCMs in the matrix.

### 3.2. Consistency Analysis

**Figure 5:**
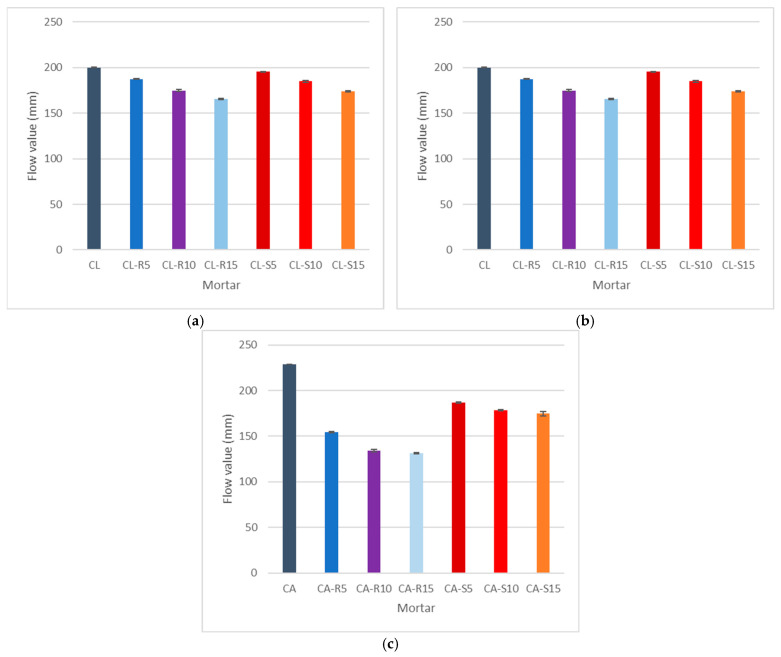
Flow table test of (**a**) mortar with Portland cement, (**b**) mortar with Portland cement and lime, and (**c**) mortar with Portland cement and APA. Vertical bears indicate standard deviation.

Figure 5 illustrates the results of the consistency tests conducted using a flow table.

Substituting cement with SCBA or RHA in cement mortar has a relatively minor impact on its consistency. The results indicate that the introduction of larger amounts of biomass leads to a slight reduction in flow. Specifically, the consistency values for the samples labelled C-R15 and C-S15 decrease by approximately 10% and 12%, respectively. The inclusion of biomass ash in cement results in an increase in carbon content, which increases the quantity of water needed to reach an acceptable level of workability [61]. In cement–lime mortar samples that incorporate biomass ashes, the flow values exhibit a consistent decrease with each incremental addition of biomass. This decrease in flow can be attributed to the inherent properties of cement–lime mortar, which tends to demand more water due to its porosity [62]. The implementation of biomass ash compounds this effect by absorbing water, further reducing the flow. It is noted that for higher replacement levels, specifically up to 15% of biomass, the reduction in flow is substantial. For example, the flow values for CL-R15 and CL-S15 decrease by approximately 20% and 15%, respectively. Additionally, the lower water-to-binder ratio in lime mortar could contribute to the observed decrease in flow values. However, it is important to note that despite these reductions, the flow values remain within the established standard requirements.

With a lower water–binder ratio, the APA cement sample exhibits a higher amount of flow, which is 26% and 20% higher than cement mortar and cement–lime mortar, respectively. This notable increase can be attributed to the incorporation of the APA, which possesses the characteristic of plasticising the mixture to achieve a more uniform and easily flowable paste [63]. Addition of RHA and SCBA to mortar causes a decrease in flow value, which can be attributed to their possible greater water absorption properties in comparison to OPC. Due to its specific properties, modified cement necessitates a higher water content compared to OPC in order to achieve saturation. Consequently, achieving the standard consistency level required a greater amount of water for modified cement compared to the reference sample [64]. The consistency of APA cement was determined to be 240 mm, and the values for 15% RHA and 15% SCBA are 140 mm and 180 mm, respectively, meaning that there is a decrease of up to 40% and 22%.

**Figure 6:**
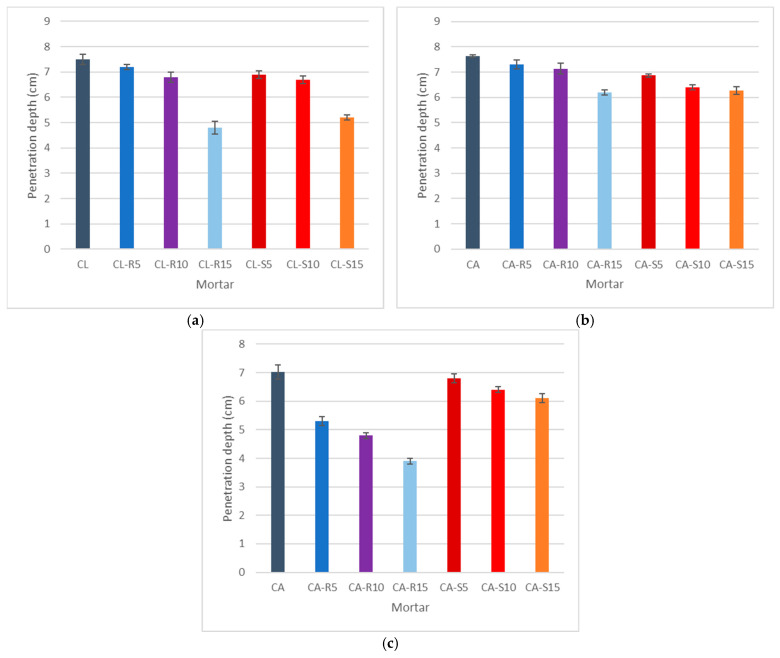
Novikov’s cone test of consistency of (**a**) mortar with Portland cement, (**b**) mortar with Portland cement and lime, and (**c**) mortar with Portland cement and APA. Vertical bears indicate standard deviation.

Secondly, the fresh mortar’s consistency, as evaluated by the cone penetrometer (Novikov’s cone), is depicted in Figure 6.

The consistency of the reference sample remains constant at 80 mm ± 5 mm, according to the established value. This predefined consistency served as the basis for determining the amount of water within the mortar composition, with the aim of achieving uniform consistency across all samples. A distinct contrast is evident when comparing the two methods to measure mortar mixture consistency. In Novikov’s cone analysis, the consistency of the mixture containing RHA decreases considerably as the replacement percentage rises. However, in the mixture with SCBA, the consistency also decreases, albeit to a lesser degree. This discrepancy can be attributed to differences in the fineness and specific area of the two biomass additives. SCBA has higher fineness, which contributes to its relatively consistent values. In contrast, RHA particles exhibit coarser characteristics, leading to increased water absorption and, subsequently, to reduced consistency. The value decreases to 42% and 20% for a higher replacement of 15% in RHA and SCBA, respectively.

In cement–lime samples, no significant differences in consistency values are observed when replacing up to 10% with either of the ash additives. However, a notable decrease in consistency is evident, reaching 37% and 35% reduction for samples CL-R15 and CL-S15, respectively, when the replacement level increases to 15%. This outcome indicates that higher levels of replacement have a pronounced impact on reducing consistency in these cement–lime samples. Similarly, the presence of biomass ashes in APA samples results in filler effects, causing consistency values to be comparable to or slightly lower than the reference sample. With a higher percentage of replacement, the consistency decreases to 5% in both cases of RHA and SCBA.

### 3.3. Compressive Strength

#### 3.3.1. Compressive Strength of Mortars with RHA Addition

**Figure 7:**
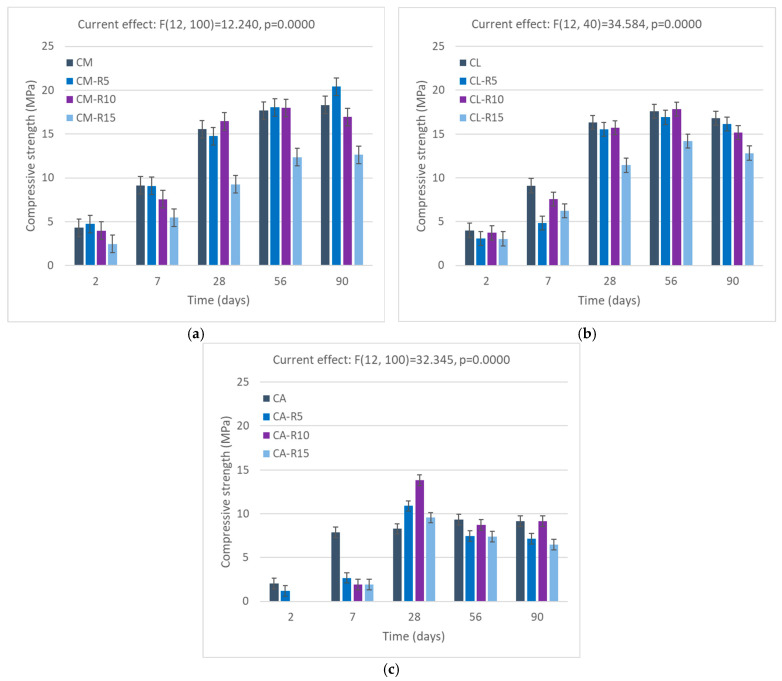
Compressive strength of RHA: (**a**) cement mortar, (**b**) lime–cement mortar, and (**c**) APA cement mortar. Error bars indicate 0.95 confidence intervals.

The results of the compressive strength tests can be seen in Figure 7 with replacement of RHA. As can be noticed, the results of the ANOVA point to the fact that the amount of RHA affects the strength of the sample. Figure 6a illustrates that with replacement at a level of 10%, rice husk ash (RHA) performed consistently better than CM in terms of strength development at the age of 28 days. The strength of RHA increased by 10% and remained constant. Incorporation of 5% rice husk ash (RHA) demonstrated a greater improvement in compressive strength, revealing a 15% improvement compared to the control sample at 90 days, highlighting the delayed pozzolanic activity of RHA, typically associated with a reduction in early-age compressive strength [65]. With the addition of 15% RHA, the compressive strength remained significantly lower than that of the reference sample, indicating that at this level of replacement, the dilution effect dominates. When the strength activity index (SAI) of cement mortars is calculated for 90 days of curing, the 5% replacement rate shows a significant improvement (SAI = 114%), while at the 10% replacement rate the change is not significant (SAI = 95%), and for 15%, the decrease is stark (SAI = 74%). It should be noted that RHA was coarse, with its fineness lower than that of cement, which could affect the pozzolanic activity, which was strongly related to the grinding and thus the fineness of the material [66].

The use of 10% RHA in the cement–lime sample did not produce significant improvements in early-age strength. However, after 56 days, there was a slightly positive impact, with the strength exceeding the control by 3%. Similar effects can be observed on the increase in strength at 56 days, as explained above in the cement mortar sample. The reactivity of RHA is beneficial on the following days of curing due to its hydration process. However, what can also be observed is a decrease in strength from after 56 days of curing. This may be related to shrinkage strain, which has been observed to occur in mortars with lime after 28 or more days [67]. This effect would require further research into the chemical reactions in the system of cement–lime–pozzolanic material at 56 days and more.

The compressive strength is lower in the APA sample compared to the other two reference samples; however, replacement of RHA in the CA samples shows beneficial results in strength with later curing days: there is a 50%, 15%, and 8% increase in the strength of CA-R10, CA-R5, and CA-R15, respectively. The improvement in compressive strength in mortar mixes with RHA can be attributed to both chemical and physical factors. Chemical effects, such as pozzolanic reactions, and physical effects, such as the filler properties of RHA, contribute to these improvements. As the use of APA increases, the air voids in the mix are further filled in by RHA components.

It should also be noted that generally, the strength of the mortars is high for masonry mortars or plasters. It reaches 16 MPa after 28 days, and can exceed 20 MPa in some cases, and the requirements for even high-strength masonry mortars Type S (ASTM C 270 [68]) provide a minimum compressive strength of 12.5 MPa after 28 days. According to European technical directives and standards, while mortars of compressive strength of 20 MPa and higher have their classification, recommendations do not contain mortars with strength of more than 20 MPa [69]. This indicates that even with the higher amounts of RHA, the mortar could be viable for use in a lower class of strength than the reference sample.

#### 3.3.2. Compressive Strength of Mortars with SCBA Addition

**Figure 8:**
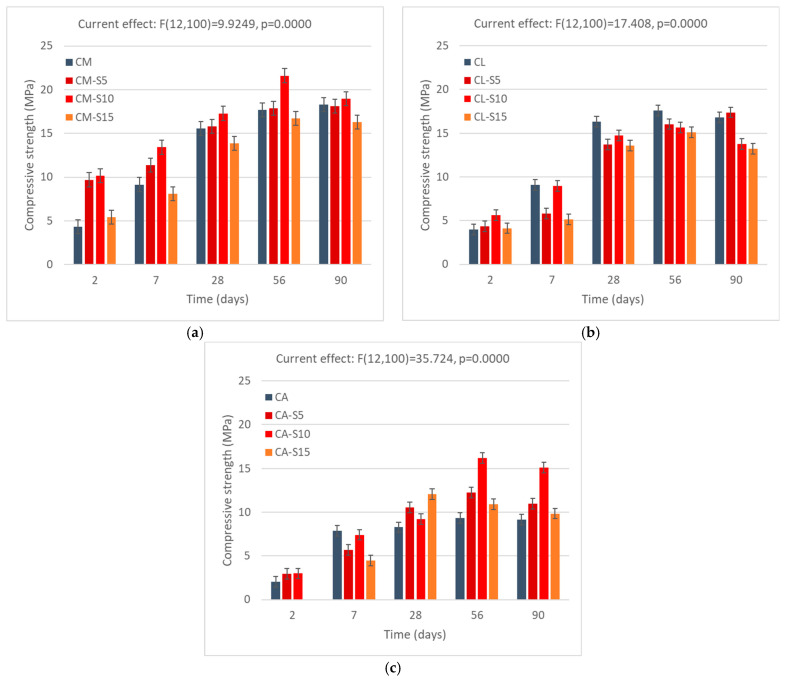
Compressive strength of mortars with SCBA: (**a**) mortar with cement, (**b**) mortar with lime–cement, and (**c**) mortar with cement and APA. Error bars indicate 0.95 confidence intervals.

Figure 8 shows the results of the compressive strength test for all SCBA percentages. Similar to the case of the RHA, the results of the ANOVA indicate that the change in the amount of SCBA affects the properties of mortars. The use of SCBA in the mortar demonstrates exceptional performance when SCBA is used as a partial replacement for cement in the cement mortar. This is due to the significant amount of amorphous silica in SCBA, which leads to its excellent filler effects. The addition of SCBA to cement mortar results in a significant improvement in compressive strength during the initial stages. The values increase by 55% and 60% after 2 days, and by 25% and 35% after 7 days, with replacement values of 5% and 10%, respectively. Strength remained constant over time compared to the reference sample and compressive strength increased to 12%, 25%, and 5% with a replacement quantity of 10% at 28, 56, and 90 days. A similar observation is made with a 10% sugarcane bagasse ash, which exhibits a compressive strength of 23.64 MPa, surpassing the 20.78 MPa of the reference mortar [70]. This means that the pozzolanic reaction occurred in which the amorphous silica in the material interacts with CH, improving the production of C–S–H and augmenting strength [71]. However, the strength activity index calculated for cement mortar samples cured for 90 days indicates little change, with the SAI of the sample with a 5% replacement rate being 98%, the 10% replacement rate—106%, and 89% for the 15% replacement rate.

In the cement–lime mortar, replacement of cement with SCBA does not lead to such improvements in strength, as the compressive strength is comparable or just slightly higher (1–2 MPa) and the strength development is stunted in relation to reference samples. The cause could be the increase in water demand in the cement–lime mixture, which affects the hydration process, as well as the potentially reduced early-age pozzolanic activity of SCBA. Although SCBA can contribute to strength development in later stages due to its pozzolanic properties, its incorporation into mortar may initially delay cement hydration and early strength gain as a result of its diluting impact [72]. After a curing time of 28 days, compressive strength results were reduced by 10% to 20%. Additionally, for the amounts of 10% and 15% SCBA addition, the strength increased from 28 to 56 days; however, after 90 days, it decreased back to the level from 28 days. As was said previously, this may be related to shrinkage strain, which has been observed to occur in mortars with lime after 28 or more days.

Adding APA to cement mortar results in the creation of more voids. SCBA particles fill these voids due to their filler properties, resulting in a higher compressive strength compared to the reference APA mortar. Due to significant water absorption, the APA samples crack during the first days of curing. However, SCBA samples increase the strength to 10%, 6%, and 25% at 28 days, 15%, 55%, and 12% at 56 days, and 8%, 40%, and 3% at 90 days with amounts of 5%, 10%, and 15%, respectively. This work aligns with other literature on the topic [73,74,75].

Similar to the case of RHA, the strength of the mortars significantly exceeds that of regular masonry mortars or plasters, and thus it may be possible for higher amounts of material to be used, with the decrease in strength placing it just in the lower strength class but still maintaining its usefulness in terms of practical application.

### 3.4. Flexural Strength Analysis

#### 3.4.1. Flexural Strength of Mortars with RHA Addition

**Figure 9:**
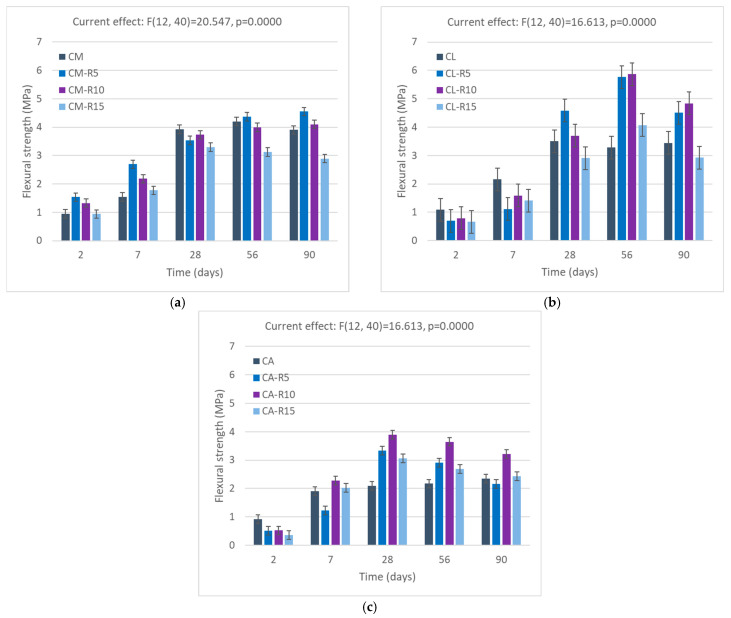
Flexural strength of mortars with RHA: (**a**) cement mortar, (**b**) lime–cement mortar, and (**c**) APA cement mortar. Error bars indicate 0.95 confidence intervals.

Figure 9 shows the flexural strength of the RHA-blended cement mortar. Comparison of the values for 2, 7, 28,56, and 90 days of strength shows that replacement with RHA in cement mortar increases flexural strength at the initial 2 and 7 days with a 10% and 25% improvement in strength. At 28 days, the strength decreases compared to the reference sample; however, with more days, there is an improvement in strength to 15% and 5% at 90 days for samples C-RHA5 and C-RHA10, respectively. Unfortunately, with a higher percentage replacement of 15%, a decrease in flexural strength can be observed.

In lime–cement mortar, replacing cement with RHA leads to improved flexural strength on each curing day. This might be due to the effects of lime as a plasticising agent that improves the surface of the mortar by reducing the volume of voids. The flexural strength increases to 20% and 5% at 28 days, 55% and 57% at 56 days, and 19% and 28% at 90 days with a replacement amount of 5% and 10%, respectively. In the APA cement samples, the replacement of cement with RHA shows a significant improvement in strength, even with a higher replacement amount of 15%, which is not common with the other two samples. The reason is that RHA increases the demand for water in the paste, and adding APA gives aerating effects to make a homogeneous paste with better consistency, which can ultimately be seen in its strength properties [60].

#### 3.4.2. Flexural Strength of Mortars with SCBA Addition

**Figure 10:**
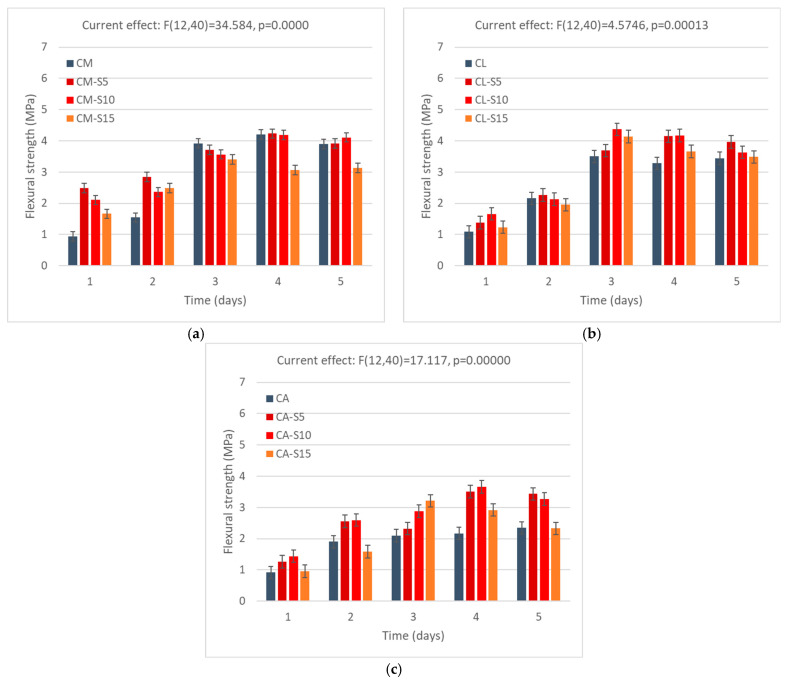
Flexural strength of SCBA mortars with (**a**) cement mortar, (**b**) lime–cement mortar, and (**c**) APA cement mortar. Error bars indicate 0.95 confidence intervals.

The results of the tests of flexural strength of mortars with SCBA addition are shown in Figure 10.

The findings of flexural strength with SCBA are shown in the Figure 10. Inclusion of SCBA in cement mortar improves flexural strength from the first days to seven days. Strength improved by 50%, 48%, 35%, and 40%, 25%, and 28% with 5%, 10%, and 15% replacement at 2 and 7 days, respectively. The strength continues to remain constant to the reference mortar for amounts of 5% and 10%, while with higher replacement, the strength decreases to 20%. The work is aligned with the experimental investigation on M30 concrete with a substitution of 5% to 20% cement with SCBA. The test results indicated that the 10% SCBA replacement produced the highest flexural strength [76], reporting a reduction in flexural strength when the proportion of SCBA in the concrete exceeded 10%. Similar to this, the authors of [77] examined the tensile strength of concrete and reported that the incorporation of SCBA (15–40%) decreased the strength.

In cement–lime mortar, SCBA addition improves flexural strength, most likely due to the pozzolanic activity of SCBA and its capacity to occupy voids within the cement matrix [78]. Maximum strength increases of 25% and 35% can be seen in samples with 5% and 10% replacement compared to the controlled samples. This may be due to the presence of SCBA. When replacing the cement with SCBA, it is found to be equal to or slightly less than that of the control mortar [31].

In APA samples, SCBA shows an increase in flexural strength compared to the other two reference samples. This improvement can be attributed to the well-known characteristics of the APA to generate air bubbles, leading to the formation of air voids. The filler properties of SCBA effectively compensate for these voids within the matrix, thus enhancing the overall strength of the samples. Across all replacement levels, flexural strength increases consistently with each day of curing, reaching a maximum improvement of 45% at the 10% replacement level.

### 3.5. Drying Shrinkage of Mortars

**Figure 11:**
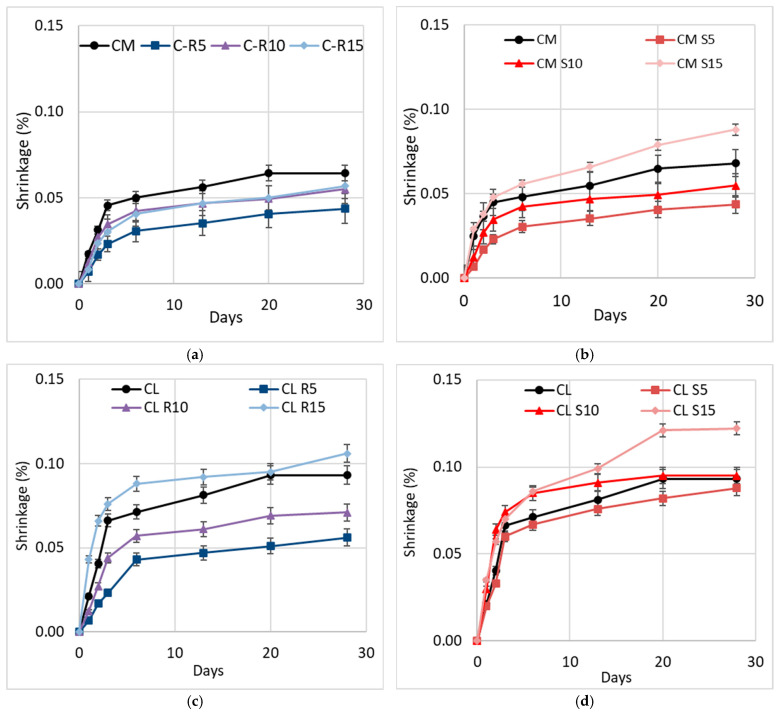
Results of shrinkage of (**a**) RHA cement mortar, (**b**) SCBA cement mortar, (**c**) RHA lime–cement mortar, and (**d**) SCBA lime–cement mortar.

Figure 11 illustrates the drying shrinkage of the mortar when RHA and SCBA are used as partial replacements for cement. When comparing RHA replacement with the reference cement mortar, it is seen that C-R5 leads to a decrease in dry shrinkage of 6%. When considering the replacement of SCBA, there is not a significant disparity in shrinkage reduction compared to the reference cement mortar. Increasing the amount of SCBA does not affect shrinkage. Subsequently, the micropore size of the mix and the pozzolanic reaction between SCBA and cement decreased, leading to a positive consequence in terms of reducing the dry shrinkage of the mortar, as seen from the MIP data provided below.

In the cement–lime mortar, replacing up to 10% of the RHA content has a positive impact on shrinkage values. The shrinkage values decrease to 35% when CL-R5 is used and to 30% when CL-R10 is used. Sample CL-S5 shows a reduction in the shrinkage value of 10%, while sample CL-S10 has an equivalent shrinkage amount to the controlled lime–cement mortar. However, when substituting for the higher value of 15%, the shrinkage increases in the case of both RHA and SCBA. Although the addition of SCMs was previously found to decrease the shrinkage [79], in this case the increase may be connected with the significant excess of water (*w*/*b* close to 1) absorbed on the material and then released as pozzolanic hydration occurs. This increase also indicates the possibility of late-age shrinkage occurring, as the shrinkage does not appear to be settled; however, this requires further research into the development of late-age shrinkage in cement–lime mortars.

### 3.6. MIP Pore Structure Analysis

**Figure 12:**
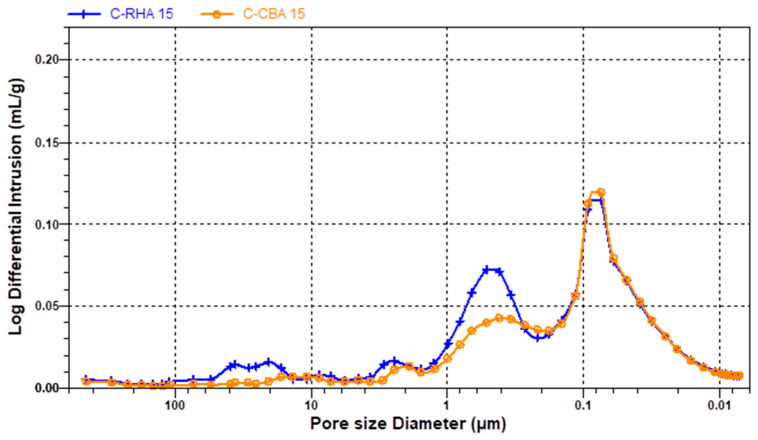
Pore structure distributions in cement mortar with RHA ashes at 15%: C-R15, and with SCBA ashes at 15%: C-S15.

**Figure 13:**
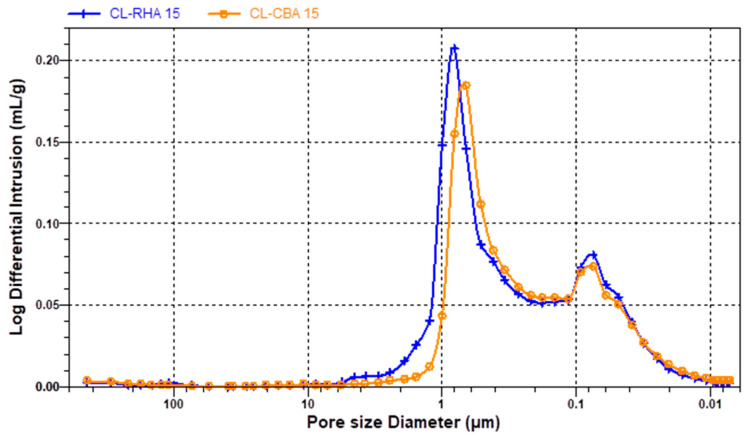
Pore structure distributions in cement–lime mortar with RHA ashes at 15%: CL-R15, and with SCB ashes at 15%: CL-S15.

**Figure 14:**
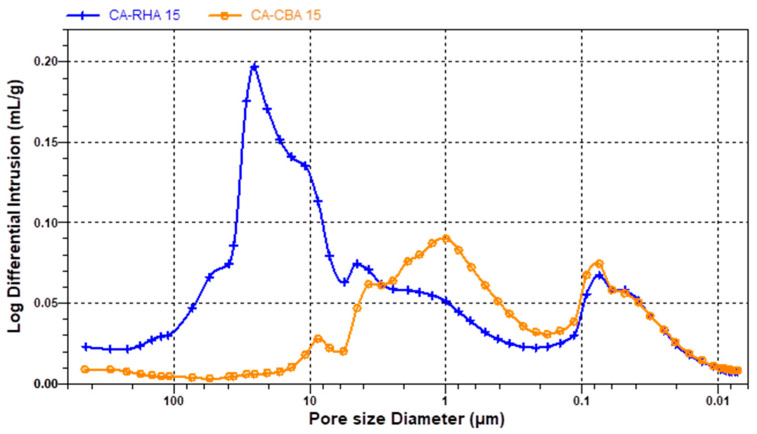
Pore structure distributions in cement mortar with APA and RHA ashes at 15%: CA-R15, and with SCB ashes at 15%: CA-S15.

Figure 12, Figure 13 and Figure 14 show the results of the MIP characteristics and the pore size distributions for the samples with 15% cement replacement. Analysis of the porosimetry data indicates that the inclusion of RHA and SCBA had distinct effects on the structure of the mortar. Specifically, the addition of RHA resulted in an increase in the proportion of pores with larger diameters that vary in the range 0.5–1.0 µm in cement and lime–cement mortars (Figure 12 and Figure 13). The inclusion of SCBA resulted in a rise in the number of pores, indicating reduced dimensions. This could be due to the greater fineness of sugarcane bagasse ash than rice husk ash (Table 2). Cement with APA has larger pores, ranging from 0.05 to 10 µm in the case of SCBA ash addition and 1 to 30 µm in the case of RHA ash addition (Figure 14). The admixture’s air-entraining effects can explain this observation. When a recently prepared mortar is exposed to water, the admixture can begin to foam and cause larger pores. The larger diameters of the pores in CA mortar with RHA ash can be caused by the lower fineness of the rice husk ash. However, SCBA reduces the pore size to some extent because SCBA, a highly pozzolanic substance characterised by its tiny particle size and high silica content, combines with calcium hydroxide to produce supplementary calcium silicate hydrate (C–S–H) gel. The subsequent C–S–H formation efficiently occupies capillary pores, thus reducing the size of the pores and enhancing the microstructure [80].

MIP analyses have also shown that the addition of RHA and SCBA ash to cement–lime mortars results in an increase in the average pore radius, specifically 0.43 and 0.33 µm, respectively. However, the inclusion of RHA or SCBA ash in cement mortars decreases the radius to 0.16 µm or 0.10 µm, respectively. In contrast, when CA mortar was mixed with APA, the inclusion of RHA ash led to an increase in the average radius of pores to 8.80 µm, but the inclusion of SCBA ash caused a notable decrease to 0.71 µm and corresponds to the different pore distributions. To sum up the discussion of the MIP results, it can be said that SCBA has more beneficial effects as an additive in mortar than RHA, since due to its surface area it reduces the pore size and porosity (28%), in comparison to RHA ash (39%), hence leading to an improvement in the mechanical properties of mortar.

**Figure 15:**
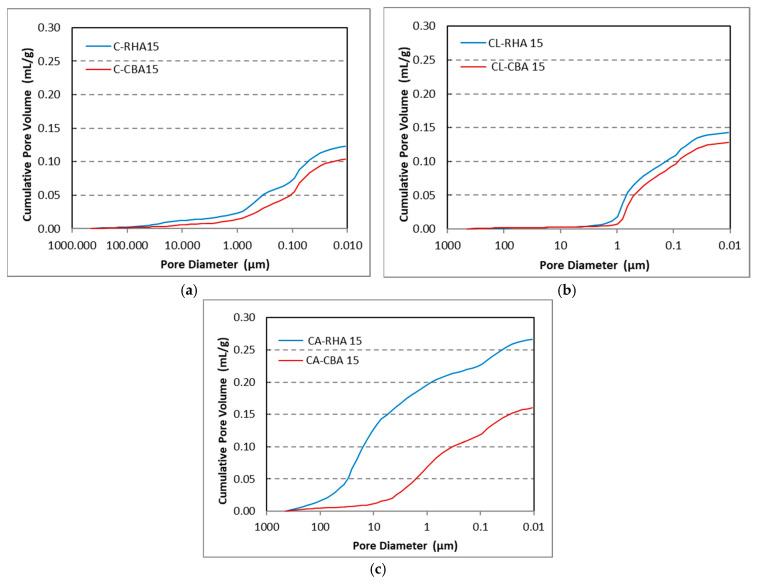
The cumulative pore size of (**a**) cement mortars, (**b**) cement–lime mortars, and (**c**) cement mortars with APA.

In Figure 15, the cumulative pore volume for the tested mortars is shown. As expected, the cumulative pore volume of the mortar with APA is highest, and cement mortar has the lowest cumulative pore volume, which is generally consistent with the results of air content.

It can be noticed that the pore volume is consistently higher for mortars with 15% RHA addition than 15% SCBA addition, regardless of mortar type, which is also consistent with air content results.

The greater pore refinement and pore volume in mortars containing SCBA are, with a high possibility, attributable to its finer particles compared to RHA, as can be ascertained by the results. However, this finding is also related to increased water absorption in SCBA resulting from its porous structure, variations in the chemical composition and reactivity rates of the materials, and possible interactions among the materials during the hydration process [81].

It should also be noted that for a significant amount of air in the APA mixtures, the number of pores in samples with SCBA is not higher than that of mortars without APA. This result is surprising; however, the answer may lie in the rheology of the SCBA mixes. SCBA mixes have shown better consistency than RHA when measured by the flow table, possibly indicating lower yield stress. This, in turn, would mean that during the process of compaction, the air enclosed in the pores of the fresh mix could more easily leave the mix, introducing a higher degree of compaction, which is consistent with the compressive strength of the samples. In the case of the addition of RHA to APA mortars, there was no possibility of conducting compressive strength testing due to the very low strength, while SCBA samples exhibited low strength at this point.

The difference in the behaviour of mortars with APA might be due to the limitation of the test itself. APA mortars are much weaker than those with no air-entraining admixture, and thus it is possible that the high pressure during the testing (>400 MPa) leads to some measure of destruction of the sample, leading to increased porosity in the samples, disproportionate to actual porosity. No signs of sample destruction were observed; however, it does not exclude the possibility of microcracks. Further testing is needed to accurately determine this phenomenon.

### 3.7. SEM Analysis Results

**Figure 16:**
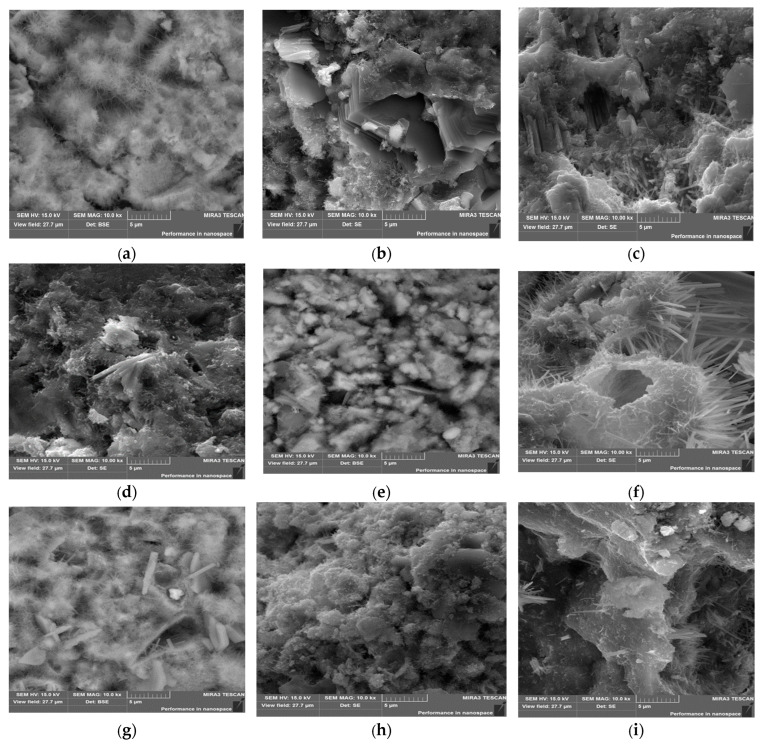
SEM micrographs for reference samples: (**a**) mortar with cement, (**b**) mortar with cement–lime, and (**c**) APA cement mortar. Samples with RHA and SCBA: (**d**) mortar with cement and 15% RHA, (**e**) mortar with lime–cement and 15% RHA, (**f**) mortar with cement, APA, and 15% RHA, (**g**) mortar with cement and 15% SCBA, (**h**) mortar with lime–cement and 15% SCBA, and (**i**) mortar with cement, APA, and 15% SCBA.

The SEM analyses of the reference sample are reported in Figure 16a–c. As can be noticed, the cement–lime mortar has a finer grain size compared to the cement mortar due to the increased presence of calcite in the matrix. When APA is added to the mortar, more well-defined “needles” can be observed (Figure 16c) in the microstructure. This effect may be due to the increased porosity of the paste, which allows for the formation and better observation of those structures in the sample. Some admixtures were also observed to promote the presence of needle-like C–S–H in the matrix [65], and it is possible that APA can affect the morphology.

Comparing the RHA samples (Figure 16d–f) to the SCBA samples (Figure 16g–i) showcases some differences that can be observed. In cement mortar, Figure 16d,g show that the RHA samples have greater voids. The fineness shows significant filler effects, resulting in a more uniform surface with reduced porosity. It should be noted that for RHA samples, there are significant differences that can be observed in the matrix between different types of mortar, possibly more pronounced than in the case of cement mortar. Lime–cement mortars with RHA exhibit a more porous matrix than cement mortars with RHA, which is consistent with research by Cizer [82], and this can be attributed to the dilution effect and the reduced amount of cement in the mortar. Although lime provides its own products, it takes longer to fully set and thus can increase porosity, which is consistent with the MIP test results. In terms of mortar with APA and RHA, it can be seen that the structure has significant amounts in the well-developed needles of C–S–H phase, which are larger than those observed for cement and cement–lime mortars with RHA. The reasons for this may be the same as in the case of mortars without SCMs, and the higher porosity (very visible in the SEM photography) is responsible for the less dense structure, allowing for this kind of development of the structure as well as to promote the presence of needle-like hydration products.

SEM photos of samples with SCBA are shown in Figure 16g–h. For the cement mortar, needle structures are more visible than in case of the reference sample and the cement mortar with RHA; however, the size of the needles is much smaller than those observed for all mortars with APA. Although this indicates an effect of SCBA that requires further testing, it also shows that the effect of APA on microstructure is significant. The addition of lime and SCBA to the mortar results in a smoother, less porous surface compared to the RHA samples. It is consistent with the results of the compressive strength and flexural strength tests, as the samples with ashes and lime have higher strength than those with cement mortar. As in previous cases, the incorporation of APA to the cement and ash matrix created larger needle-shaped crystals of C–S–H.

## 4. Conclusions

This research provided insights into how natural plant waste ashes affect the physical, mechanical, and microstructural properties of cement mortar with variable binder additions. The following conclusions were obtained based on the experimental results:

- Air content was affected to a small degree by the presence of SCBA and RHA, and the air content generally increased with the increase in SCM content. This may be due to the irregular shapes of the grains. In APA mortar samples, air voids arose due to the aerating effect of the admixture, and in this case no significant effect of the SCMs could be observed, possibly due to the large amount of air voids offsetting the issues caused by the shape of grains and changes in consistency.
- The consistency by flow table analysis showed that replacement of 15% decreased the flow values of mixtures for both RHA and SCBA by 10% and 12% in cement mortar, 20% and 15% for cement–lime mortar, and 40% and 22% for APA cement mortar, respectively. Similar results were obtained for Novikov’s cone test. This effect may be linked to the fact that SCM addition increases the water demand on mixtures.
- RHA addition to cement mortar enhanced the compressive strength by up to 15% at 90 days. In cement–lime samples, strength increased slightly at 56 days, then decreased in the remaining days, which may possibly be a result of delayed shrinkage strains. In APA samples, RHA addition improved compressive strength by up to 50%. Flexural strength of mortars with RHA was higher than that of the reference sample; however, after 28 days their strength was comparable. In case of lime mortars, the early strength or mortar with RHA was lower than that of the reference sample, but comparable or higher at later dates. In the APA sample, the RHA showed a significant improvement in strength even with a higher replacement amount of 15%. The positive effect of RHA on strength can be attributed to the filler effect and pozzolanic reaction of RHA.
- SCBA outperformed RHA in compressive strength of cement mortar. SCBA increased the strength of cement mortars; however, with cement–lime mortar, the compressive strength of samples with RHA remained lower until 56 days. Surprisingly, the RHA addition increased strength for APA mortars by 25%, 55%, and 40% at 5%, 10%, and 15% replacement rates. SCBA increased the flexural strength of cement mortar by 50% to 48%, 35% to 40%, and 25% to 28% with replacement by 5%, 10%, and 15% at 2 and 7 days. Strength dropped by 20% with increased replacement. In case of SCBA replacement in cement–lime mortar, an increase of flexural strength occurred. The SCBA in APA samples increased flexural strength.
- RHA replacement decreased dry shrinkage by up to 35%. SCBA additions of 10% and 15% reduced the shrinkage. Those effects may be attributed to the excess water in the mortar being absorbed on SCM grains and then released.
- Adding RHA increased the pore size (0.5–1 µm) in cement mortar and by a higher amount in cement–lime mortar in comparison to the SCBA additive. Additionally, APA increased the pore size (1–30 µm) in mortar with the RHA additive. MIP results indicated a greater share of pores with smaller diameters for the SCBA additive, which translated into higher strength results but also a tendency towards greater shrinkage in comparison with the RHA additive, possibly due to differences in the morphology of the ash particles.
- The SEM examination results showed that bagasse ash mortar, especially with lime, had a finer microstructure and was less porous than mortar with the RHA additive. In case of the APA-containing cement mortar, the microstructure was more porous due to larger voids. Mortar containing lime and biomass ash had a homogenous structure with fewer pores, which helped improve its strength.

The final conclusion can be formulated that RHA and SCBA have proven to be beneficial additives for masonry mortars due to their strength properties in a certain composition range, with limited shrinkage at lower ash contents. It has been ascertained that for both RHA and SCBA, amounts of 5–10% can be used with cement mortar, cement–lime mortar, and mortar with APA to obtain mostly similar or better results than those of reference samples in terms of consistency, shrinkage, and strength. It is, therefore, possible that they could be used in cements, especially in light of the increased emphasis on using waste material in cement production.

The conducted research indicated a need for further research to provide more in-depth knowledge about the observed phenomena. Rheological testing and XRD analysis of the mortars would be instrumental in furthering the understanding of the reactions that shape the properties of the mortars. Additionally, future directions of the tests should include the long-term testing of shrinkage and strength, as well as durability testing, as those elements are necessary to provide information about long-term use of these materials.

## Figures and Tables

**Table 1:** Chemical composition of OPC, lime, and agro-industrial wastes.

Chemical Composition	OPC (%)	Lime (%)	RHA (%)	SCBA (%)
SiO_2_	18.9	0.7	86.73	66.5
Al_2_O	3.8		0.04	4.82
Fe_2_O_3_	3.9		0.61	4.67
CaO	63.3	90.2	0.39	3.83
MgO	1.2	1	0.08	2.87
SO_3_	2.9	0.7	1.32	
Na_2_O	0.15	0.15	9.76	0.59
K_2_O	1.05		0.01	4.07

**Table 2:** Physical properties of RHA, SCBA, and OPC.

Properties	OPC	RHA	SCBA
Fineness, retained on 45 μm (%)	13	19	14
Density (kg/m^3^)	3110	2240	2200

**Table 3:** Mixture proportions of all the mortar samples.

Mixtures	Constituents (g)							
	CEM I 42.5R	Lime	Water	*w*/*b* Ratio (-)	Sand	Natural SCMs		Air-Entraining Admixture (APA)
						RHA	SCBA	
Cement mortar								
CM	450	-	440	0.98	2308	-	-	-
C-R5	427.5	-			2308	22.5	-	
C-R10	405	-			2308	45	-	
C-R15	382.5	-			2308	67.5	-	
C-S5	427.5	-			2308	-	22.5	
C-S10	405	-			2308	-	45	
C-S15	382.5	-			2308	-	67.5	
Cement–lime mortar								
CL	350	253	410	0.68	1795	-	-	-
CL-R5	332.5	253			1795	17.5	-	
CL-R10	315	253			1795	35	-	
CL-R15	297.5	253			1795	52.5	-	
CL-S5	332.5	253			1795	-	17.5	
CL-S10	315	253			1795	-	35	
CL-S15	297.5	253			1795	-	52.5	
Cement mortar with APA								
CA	450	-	310	0.69	1795	-	-	2.25
CA-R5	427.5	-			1795	17.5	-	
CA-R10	405	-			1795	35	-	
CA-R15	382.5	-			1795	52.5	-	
CA-S5	427.5	-			1795	-	17.5	
CA-S10	405	-			1795	-	35	
CA-S15	382.5	-			1795	-	52.5	

## Data Availability

The original contributions presented in this study are included in the article. Further inquiries can be directed to the corresponding author.

## References

[1] Kimbonguila A., Matos L., Petit J., Scher J., Nzikou J.-M. (2019). Effect of Physical Treatment on the Physicochemical, Rheological and Functional Properties of Yam Meal of the Cultivar ‘Ngumvu’ from *Dioscorea alata* L. of Congo. Int. J. Recent Sci. Res..

[2] Hn S., Deotale A.R., Sathawane R.S. (2012). Effect of Partial Replacement of Cement by Fly Ash, Rice Husk Ash with Using Steel Fiber in Concrete. Int. J. Sci. Eng. Res..

[3] Bui D.D., Hu J., Stroeven P. (2005). Particle size effect on the strength of rice husk ash blended gap-graded Portland cement concrete. Cem. Concr. Compos..

[4] Awad Allah Mohamed Y. (2017). Effects of Sugarcane’s Bagasse Ash Additive on Portland Cement Properties. Int. J. Sustain. Dev. Res..

[5] Bie R.-S., Song X.-F., Liu Q.-Q., Ji X.-Y., Chen P. (2015). Studies on effects of burning conditions and rice husk ash (RHA) blending amount on the mechanical behavior of cement. Cem. Concr. Compos..

[6] Zhang M.H., Lastra R., Malhotra V.M. (1996). Rice-husk ash paste and concrete: Some aspects of hydration and the microstructure of the interfacial zone between the aggregate and paste. Cem. Concr. Res..

[7] Jamil M., Khan M.N., Karim M.R., Kaish A.B., Zain M.F. (2016). Physical and chemical contributions of Rice Husk Ash on the properties of mortar. Constr. Build. Mater..

[8] Coutinho J.S., Papadakis V.G. Rice Husk Ash—Importance of Fineness for its Use as a Pozzolanic and Chloride-Resistant Material. Proceedings of the 12th International Conference o Durability of Building Materials ad Components.

[9] Feng Q.-G., Lin Q.-Y., Yu Q.-J., Zhao S.-Y., Yang L.-F., Sugita S. (2004). Concrete with highly active rice husk ash. J. Wuhan Univ. Technol. Mat. Sci. Edit..

[10] Ganesan K., Rajagopal K., Thangavel K. (2008). Rice husk ash blended cement: Assessment of optimal level of replacement for strength and permeability properties of concrete. Constr. Build. Mater..

[11] Bezerra I.M.T., Figueiredo S.S., De Carvalho J.B.Q., Neves G.A., De Souza J., Menezes R.R. (2012). Coating mortar using rice husk ash as binding. Mater. Sci. Forum.

[12] Mahmud H.B., Hamid N.A.A., Chin K.Y. Production of High Strength Concrete Incorporating an Agricultural Waste-Rice Husk Ash. Proceedings of the 2010 2nd International Conference on Chemical, Biological and Environmental Engineering.

[13] Barbhuiya S., Das B.B., Adak D., Rajput A., Katare V. (2025). Rice Husk Ash in Structural Concrete: Influence on Strength, Durability and Sustainability. Discov. Concr. Cem..

[14] Hasan N.M.S., Sobuz M.H.R., Khan M.M.H., Mim N.J., Meraz M.M., Datta S.D., Rana M.J., Saha A., Akid A.S.M., Mehedi M.T. (2022). Integration of Rice Husk Ash as Supplementary Cementitious Material in the Production of Sustainable High-Strength Concrete. Materials.

[15] Van Tuan N., Ye G., Van Breugel K., Copuroglu O. (2011). Hydration and Microstructure of Ultra High Performance Concrete Incorporating Rice Husk Ash. Cem. Concr. Res..

[16] Givi A.N., Rashid S.A., Aziz F.N.A., Salleh M.A.M. (2010). Assessment of the Effects of Rice Husk Ash Particle Size on Strength, Water Permeability and Workability of Binary Blended Concrete. Constr. Build. Mater..

[17] Park K.-B., Kwon S.-J., Wang X.-Y. (2016). Analysis of the Effects of Rice Husk Ash on the Hydration of Cementitious Materials. Constr. Build. Mater..

[18] Ahmad I.A., Lopa A.T., Angraini A., Pertiwi N., Taufieq A.S. (2022). Investigation of Mortar Using Rice Husk Ash as Partial Substitution of Porland Composite Cement. IOSR J. Mech. Civ. Eng..

[19] Le H.T., Kraus M., Siewert K., Ludwig H.-M. (2015). Effect of Macro-Mesoporous Rice Husk Ash on Rheological Properties of Mortar Formulated from Self-Compacting High Performance Concrete. Constr. Build. Mater..

[20] Habeeb G.A., Fayyadh M.M. (2009). Rice Husk Ash Concrete: The Effect of RHA Average Particle Size on Mechanical Properties and Drying Shrinkage. Aust. J. Basic Appl. Sci..

[21] Huang H., Ye G. Use of Rice Husk Ash for Mitigating the Autogenous Shrinkage of Cement Pastes at Low Water Cement Ratio. Proceedings of the Ultra-High Performance Concrete and Hgh Performance Construction Materials.

[22] Yaseen N. (2024). Exploring the Potential of Sugarcane Bagasse Ash as a Sustainable Supplementary Cementitious Material: Experimental Investigation and Statistical Analysis. Results Chem..

[23] Dos Santos F.C., Silva Américo R.M. (2024). Application of sugarcane bagasse ash in mortars: Systematic literature review. Rev. Tec..

[24] Francioso V., Lemos-Micolta E.D., Elgaali H.H., Moro C., Rojas-Manzano M.A., Velay-Lizancos M. (2024). Valorization of Sugarcane Bagasse Ash as an Alternative SCM: Effect of Particle Size, Temperature-Crossover Effect Mitigation & Cost Analysis. Sustainability.

[25] Arif E., Clark M.W., Lake N. (2016). Sugar cane bagasse ash from a high efficiency co-generation boiler: Applications in cement and mortar production. Constr. Build. Mater..

[26] Ganesan K., Rajagopal K., Thangavel K. (2007). Evaluation of bagasse ash as supplementary cementitious material. Cem. Concr. Compos..

[27] Ahmad W., Ahmad A., Ostrowski K.A., Aslam F., Joyklad P., Zajdel P. (2021). Sustainable approach of using sugarcane bagasse ash in cement-based composites: A systematic review. Case Stud. Constr. Mater..

[28] Jha P., Sachan A.K., Singh R.P. (2021). Agro-waste sugarcane bagasse ash (ScBA) as partial replacement of binder material in concrete. Mater. Today Proc..

[29] Moraes J.C.B., Akasaki J.L., Melges J.L.P., Monzó J., Borrachero M.V., Soriano L., Payá J., Tashima M.M. (2015). Assessment of sugar cane straw ash (SCSA) as Pozzolanic material in blended portland cement: Microstructural characterization of pastes and mechanical strength of mortars. Constr. Build. Mater..

[30] Le D.-H., Sheen Y.-N., Lam M.N.-T. (2018). Fresh and hardened properties of self-compacting concrete with sugarcane bagasse ash–slag blended cement. Constr. Build. Mater..

[31] Modani P.O., Vyawahare M.R. (2013). Utilization of bagasse ash as a partial replacement of fine aggregate in concrete. Procedia Eng..

[32] Mangi S.A., Jamaluddin N., Wan Ibrahim M.H., Abdullah A.H., Abdul Awal A.S.M., Sohu S., Ali N. (2017). Utilization of sugarcane bagasse ash in concrete as partial replacement of cement. IOP Conf. Ser. Mater. Sci. Eng..

[33] Batool F., Masood A., Ali M. (2020). Characterization of Sugarcane Bagasse Ash as Pozzolan and Influence on Concrete Properties. Arab. J. Sci. Eng..

[34] Chusilp N., Jaturapitakkul C., Kiattikomol K. (2009). Utilization of bagasse ash as a pozzolanic material in concrete. Constr. Build. Mater..

[35] He J., Kawasaki S., Achal V. (2020). The Utilization of Agricultural Waste as Agro-Cement in Concrete: A Review. Sustainability.

[36] Cordeiro G.C., Andreão P.V., Tavares L.M. (2019). Pozzolanic Properties of Ultrafine Sugar Cane Bagasse Ash Produced by Controlled Burning. Heliyon.

[37] Maghraby Y.E., Talaat M., Zenhom M., Moussa R.R., Salem S. (2025). Utilization of Untreated Sugarcane Bagasse Ash in the Construction Industry. J. Umm Al-Qura Univ. Eng. Archit..

[38] Kolawole J.T., Babafemi A.J., Fanijo E., Chandra Paul S., Combrinck R. (2021). State-of-the-Art Review on the Use of Sugarcane Bagasse Ash in Cementitious Materials. Cem. Concr. Compos..

[39] Singh N.B., Singh V.D., Rai S. (2000). Hydration of Bagasse Ash-Blended Portland Cement. Cem. Concr. Res..

[40] Rossignolo J.A., Rodrigues M.S., Frias M., Santos S.F., Junior H.S. (2017). Improved Interfacial Transition Zone between Aggregate-Cementitious Matrix by Addition Sugarcane Industrial Ash. Cem. Concr. Compos..

[41] Chi M.-C. (2012). Effects of Sugar Cane Bagasse Ash as a Cement Replacement on Properties of Mortars. Sci. Eng. Compos. Mater..

[42] Andrade Neto J.D.S., De França M.J.S., Amorim Júnior N.S.D., Ribeiro D.V. (2021). Effects of adding sugarcane bagasse ash on the properties and durability of concrete. Constr. Build. Mater..

[43] Cordeiro G.C., Toledo Filho R.D., Tavares L.M., Fairbairn E.M.R. (2008). Pozzolanic Activity and Filler Effect of Sugar Cane Bagasse Ash in Portland Cement and Lime Mortars. Cem. Concr. Compos..

[44] Malathy R., Shanmugam R., Chung I.-M., Kim S.-H., Prabakaran M. (2022). Mechanical and Microstructural Properties of Composite Mortars with Lime, Silica Fume and Rice Husk Ash. Processes.

[45] Cook D.J., Pama R.P., Paul B.K. (1977). Rice Husk Ash-Lime-Cement Mixes for Use in Masonry Units. Build. Environ..

[46] Nayak J.R. (2023). Analysis of Impact of Selected Natural Waste Fibers and Ashes on Properties of Mortars. Ph.D. Thesis.

[47] (2016). Specification for Mortar for Masonry. Rendering and Plastering Mortar.

[48] Jittin V., Minnu S.N., Bahurudeen A. (2021). Potential of sugarcane bagasse ash as supplementary cementitious material and comparison with currently used rice husk ash. Constr. Build. Mater..

[49] Xu W., Lo T.Y., Memon S.A. (2012). Microstructure and reactivity of rich husk ash. Constr. Build. Mater..

[50] Jiménez-Quero V.G., Ortiz-Guzmán M., Montes-García P. (2019). Durability of mortars containing sugarcane bagasse Ash. J. Phys. Conf. Ser..

[51] (2011). Cement—Part 1: Composition, Specifications and Conformity Criteria for Common Cements.

[52] (2016). Masonry Cement—Part 2: Test Methods.

[53] (1985). Zaprawy Budowlane. Badanie Cech Fizycznych i Wytrzymałościowych (Construction Mortars. Testing of Physical and Strength Properties).

[54] (2000). Methods of Test for Mortar for Masonry—Part 3: Determination of Consistence of Fresh Mortar (by Flow Table).

[55] (2001). Methods of Test for Mortar for Masonary—Part 11: Determination of Flexural and Compressive Strength of Hardened Mortar.

[56] (2004). Test Methods—Part 4: Determination of Shrinkage and Elongation.

[57] Neville A.M., Brooks J.J. (2010). Concrete Technology.

[58] Saloni, Parveen, Pham T.M., Lim Y.Y., Pradhan S.S., Jatin, Kumar J. (2021). Performance of Rice Husk Ash-Based Sustainable Geopolymer Concrete with Ultra-Fine Slag and Corn Cob Ash. Constr. Build. Mater..

[59] Promsawat P., Chatveera B., Sua-iam G., Makul N. (2020). Properties of self-compacting concrete prepared with ternary Portland cement-high volume fly ash-calcium carbonate blends. Case Stud. Constr. Mater..

[60] Gołaszewska M., Gołaszewski J., Bochen J., Cygan G. (2022). Comparative Study of Effects of Air-Entraining Plasticizing Admixture and Lime on Physical and Mechanical Properties of Masonry Mortars and Plasters. Materials.

[61] Vu V.-A., Cloutier A., Bissonnette B., Blanchet P., Duchesne J. (2019). The Effect of Wood Ash as a Partial Cement Replacement Material for Making Wood-Cement Panels. Materials.

[62] Gulbe L., Vitina I., Setina J. (2017). The Influence of Cement on Properties of Lime Mortars. Procedia Eng..

[63] Rashmi Nayak J., Bochen J., Gołaszewska M. (2022). Experimental studies on the effect of natural and synthetic fibers on properties of fresh and hardened mortar. Constr. Build. Mater..

[64] Nath Bhowmik R., Pal J. (2023). Application of Consistency-Based Water-to-Binder Ratio to Compensate Workability Loss in Concrete Modified with Rice Husk Ash. Mater. Today Proc..

[65] De Silva G.H.M.J.S., Naveen P. (2024). Effect of rice husk ash and coconut coir fiber on cement mortar: Enhancing sustainability and efficiency in buildings. Constr. Build. Mater..

[66] Siddika A., Mamun M.A.A., Alyousef R., Mohammadhosseini H. (2021). State-of-the-Art-Review on Rice Husk Ash: A Supplementary Cementitious Material in Concrete. J. King Saud Univ. Eng. Sci..

[67] Pozo-Antonio J.S. (2015). Evolution of Mechanical Properties and Drying Shrinkage in Lime-Based and Lime Cement-Based Mortars with Pure Limestone Aggregate. Constr. Build. Mater..

[68] (2024). Standard Specificafion for Mortar for Unit Masonry.

[69] Stowarzyszenie Przemysłu Wapienniczego Tradycyjne Zaprawy Murarskie i Tynkarskie (Traditional Masonry Mortars and Plasters). https://wapno-info.pl/wp-content/uploads/2022/11/112957-tradycyjne-zaprawy-murarskie-i-tynkarskie.pdf.

[70] Cupim R.V., Tostes Linhares Júnior J.A., Mesquita L.C., Marques M.G., Garcez de Azevedo A.R., Marvila M.T. (2025). Rheological and Mechanical Properties of Mortars Made with Recycled Sugarcane Bagasse Ash. J. Mater. Res. Technol..

[71] Liew Y.X., Saad S.A., Anand N., Tee K.F., Chin S.C. (2024). Evaluating the Impact of Reducing POFA’s Particle Fineness on Its Pozzolanic Reactivity and Mortar Strength. J. Mater. Sci. Mater. Eng..

[72] Bahurudeen A., Kanraj D., Gokul Dev V., Santhanam M. (2015). Performance Evaluation of Sugarcane Bagasse Ash Blended Cement in Concrete. Cem. Concr. Compos..

[73] Shaban W.M., Heniegal A.M., Amin M., Zeyad A.M., Agwa I.S., Hassan H.H. (2024). Effect of Agricultural Wastes as Sugar Beet Ash, Sugarcane Leaf Ash, and Sugarcane Bagasse Ash on UHPC Properties. J. Build. Eng..

[74] Verma M., Singh K.R. (2024). Performance Characteristic of Sugarcane Fiber and Bagasse Ash as Cement Replacement in Sustainable Concrete. Innov. Infrastruct. Solut..

[75] Chusilp N., Jaturapitakkul C., Kiattikomol K. (2009). Effects of LOI of Ground Bagasse Ash on the Compressive Strength and Sulfate Resistance of Mortars. Constr. Build. Mater..

[76] Inbasekar M., Hariprasath P., Senthilkumar D. (2016). Study on potential utilization of sugarcane bagasse ash in steel fiber reinforced concrete. Int. J. Eng. Sci. Res. Technol..

[77] Jagadesh P., Ramachandra Murthy A., Murugesan R. (2020). Effect of Processed Sugar Cane Bagasse Ash on Mechanical and Fracture Properties of Blended Mortar. Constr. Build. Mater..

[78] Weng J.-R., Liao W.-C. (2021). Microstructure and Shrinkage Behavior of High-Performance Concrete Containing Supplementary Cementitious Materials. Constr. Build. Mater..

[79] Torres-Ortega R., Torres-Sanchez D., Lopez-Lara T. (2025). Mechanical Properties of Hydraulic Concretes with Partial Replacement of Portland Cement by Pozzolans Obtained from Agro-Industrial Residues: A Review. Heliyon.

[80] Fapohunda C., Akinbile B., Shittu A. (2017). Structure and Properties of Mortar and Concrete with Rice Husk Ash as Partial Replacement of Ordinary Portland Cement—A Review. Int. J. Sustain. Built Environ..

[81] Yan Y., Tang J., Geng G. (2023). Exploring Microstructure Development of C-S-H Gel in Cement Blends with Starch-Based Polysaccharide Additives. Case Stud. Constr. Mater..

[82] Cizer O., Balen K.V., Gemert D.V., Elsen J. (2007). Carbonation and Hydration of Mortars with Calcium Hydroxide and Calcium Silicate Binders. Sustainable Construction Materials and Technologies.

